# Face masks versus sunglasses: limited effects of time and individual differences in the ability to judge facial identity and social traits

**DOI:** 10.1186/s41235-022-00371-z

**Published:** 2022-02-16

**Authors:** Rachel J. Bennetts, Poppy Johnson Humphrey, Paulina Zielinska, Sarah Bate

**Affiliations:** 1 Division of Psychology, College of Health, Medicine and Life Sciences, Brunel University London, Kingston Lane, Uxbridge, UB8 3PH UK; 2 Department of Psychology, Faculty of Science and Technology, Bournemouth University, Poole, UK

**Keywords:** Face coverings, Face recognition, Trait judgements, Prosopagnosia, Super recognisers

## Abstract

Some research indicates that face masks impair identification and other judgements such as trustworthiness. However, it is unclear whether those effects have abated over time as individuals adjust to widespread use of masks, or whether performance is related to individual differences in face recognition ability. This study examined the effect of masks and sunglasses on face matching and social judgements (trustworthiness, competence, attractiveness). In Experiment 1, 135 participants across three different time points (June 2020–July 2021) viewed unedited faces and faces with masks, sunglasses, or both. Both masks and sunglasses similarly decreased matching performance. The effect of masks on social judgements varied depending on the judgement and whether the face was depicted with sunglasses. There was no effect of timepoint on any measure, suggesting that the effects of masks have not diminished. In Experiment 2, 12 individuals with developmental prosopagnosia (DP) and 10 super-recognisers (SRs) completed the same tasks. The effect of masks on identity matching was reduced in SRs, whereas the effects of masks and sunglasses for the DP group did not differ from controls. These findings indicate that face masks significantly affect face perception, depending on the availability of other facial information, and are not modified by exposure.

## Introduction

In response to the COVID-19 pandemic, use of face coverings (masks)^1^ was introduced to mitigate the spread of the disease in many countries (Felter & Bussemaker, 2020). Masks offer substantial public health benefits (Brooks & Butler, 2021; Howard et al., 2021), but there is preliminary evidence that they may impair social interactions by obscuring areas of the face that carry cues to emotions, identity, speech information, and other social judgements (Biermann et al., 2021; Carbon, 2020; Freud et al., 2020; Marini et al., 2021; Noyes et al., 2021; Saunders et al., 2021). However, the consistency of these effects over time (i.e., as people adjust to the use of masks in everyday life) and across individuals remains unclear. In this study, we examine the effects of masks on two face perception tasks: face identity matching and social judgements (trustworthiness, competence, and attractiveness), and compare them to the effects of sunglasses. Specifically, we examine (1) whether the effects of face coverings remained consistent over 13 months during the pandemic; and (2) whether the effects of face coverings on face perception are related to individual differences in face recognition ability.

### Masks and identification

There are good reasons to believe that identity matching (specifically, matching unfamiliar faces) could be adversely affected by masks. Decades of research has established that unfamiliar face matching is error-prone, and even small variations between images can reduce accuracy. For example, in simple tasks which present two images side by side and ask participants to judge whether they are the same or a different person, accuracy tends to vary from around 80–90% (e.g., Burton et al., 2010; Carragher & Hancock, 2020; Megreya & Burton, 2007). Accuracy declines further when images contain naturalistic variability (e.g., pictures taken at different time-points and in different settings; Bate et al., 2018; Fysh & Bindemann, 2018). Similarly, the addition of simple props or occlusions, such as spectacles or sunglasses, also has a negative impact on unfamiliar face matching (Graham & Ritchie, 2019; Kramer & Ritchie, 2016), particularly when only one image is displayed with eyewear.

There is comparatively less work on occlusions which, like masks, obscure the lower face. Freud et al. (2020) and Marini et al. (2021) both investigated the effects of masks on face learning. Marini et al. found that presenting faces with masks in the learning phase impeded subsequent recognition. Notably, these effects were even present when the faces were shown with transparent masks (Marini et al., 2021), supporting the claim that even subtle occlusions can impair unfamiliar face processing. Freud et al. reported similar results: they found that learning masked face impeded subsequent recognition accuracy (and vice versa).

Studies on face matching also support the claim that masks impede unfamiliar face identification. Carragher and Hancock (2020) investigated the effects of masks on simultaneous face matching, and found that both human observers and computer-based face recognition systems are negatively affected by the addition of masks to images. Notably, these effects were similar in scale regardless of whether one or both images to be matched were shown wearing a mask. Furthermore, masks also biased participants’ responding, making it more likely that they would classify unfamiliar faces as “different” or “mismatched”. Noyes et al. (2021) found similar results (although a smaller effect size) for both accuracy and bias using more naturalistic images (images sourced from the internet, rather than images with masks edited on).

Noyes et al. (2021) also examined the effects of different types of facial occlusions, and reported that the effect of masks on unfamiliar face matching was slightly larger than the effect of sunglasses. Nonetheless, accuracy in both conditions remained well above chance levels, suggesting that occlusion of any one area or feature does not abolish face recognition abilities. This is in line with research using the “bubbles” technique (which reveals areas of the face necessary for identification; Gosselin & Schyns, 2001) and research into “critical features” in face recognition (Abudarham & Yovel, 2016) which suggest that information from both the upper and lower regions of the face is important during face identification tasks. Noyes et al.’s results indicate that individuals may use this information flexibly when some areas of the face are occluded. However, it is unclear what effect multiple occlusions (i.e., sunglasses and masks together) may have on identification, particularly with more variable images (e.g., images taken with different cameras or at different timepoints).

### Face coverings and social judgements

Identity is not the only information carried in a face. People frequently make social attributions such as how trustworthy, competent, or attractive a person is based on their face, and these attributions can have consequences for a wide range of behaviours, such as behaviour in economic games, voting choices, and dating (Todorov et al., 2015).

Compared to identity, there have been fewer studies examining the effects of masks and other occlusions on social judgements. Graham and Ritchie (2019) assessed the effects of spectacles and sunglasses on social trait judgements, and found that sunglasses (but not spectacles) reduced judgements of trustworthiness, but did not affect judgements of competence or attractiveness. Information from both the eye and mouth region is involved in trustworthiness and competence judgements (Dotsch & Todorov, 2012; Olivola & Todorov, 2010; Riggio & Riggio, 2010), so it is reasonable to assume that masks could lead to similar perceptual effects. In support of this, Marini et al. (2021) found that transparent face masks did not have a significant effect on trustworthiness judgements, whereas opaque masks did affect trustworthiness judgements for some faces (specifically, they increased trustworthiness ratings for “untrustworthy” faces) (see also Biermann et al., 2021). Attractiveness judgements are influenced by facial symmetry (Rhodes, 2006) and contrast, particularly in the eye region (Killian et al., 2018). Consequently, the potential effects of masks on attractiveness judgements are unclear: they may occlude some important cues to attractiveness by making symmetry judgements more difficult, whilst leaving other cues available.

Thus, there is good reason to believe that face coverings, including masks and sunglasses, affect identification and social judgements. However, less is known about whether and how this varies over time.

### Variability of effects over time

Much of the data about masks and face processing reported to date was collected in the early months of the pandemic—for example, Biermann et al. (2021) collected data between July and October 2020, when masks had been mandatory for three to six months in Germany (where the data was collected). At this point, most individuals in Western countries had relatively limited exposure to masks, so it is unsurprising that this unfamiliar perceptual occlusion disrupted face perception. However, there is some evidence that face perception can adapt to perceptual input over time. For example, own-group biases (e.g., the own-race effect, own-age effect) are a phenomenon whereby people show poorer performance when identifying faces of a different social group than their own (Anastasi & Rhodes, 2005; Meissner & Brigham, 2001). These biases are often attributed to a lack of perceptual experience with the “out-group”. However, prolonged contact with the “out-group” (Hancock & Rhodes, 2008; Harrison & Hole, 2009) and training programmes focused on individuating out-group faces (Tanaka & Pierce, 2009) can ameliorate these biases, indicating that exposure and training may mitigate perceptual limitations in our face processing system.

It is possible that the same processes could act to mitigate or compensate for the effects of masks on identification. In support of this, a very recent paper found that training individuals to focus on diagnostic features (e.g., ears or visible marks) could improve covered face recognition (Carragher et al., 2021). It is possible that, following prolonged periods of exposure to masks (i.e., in the 12–16 months since face coverings were mandated in certain areas of the UK), individuals could adapt or develop their own strategies which could reduce the effects of masks on identification. A change in effects over time could also explain why previous studies (Carragher & Hancock, 2020; Noyes et al., 2021) found different effect sizes for unfamiliar face matching, despite the use of relatively similar tasks.

Opinions on masks and the proportion of people wearing them regularly have also varied over time (Nolsoe, 2021; Smith, 2020), which raises the possibility that social judgements about mask-wearers could also vary depending on the context at the time of data collection. However, no research to date has attempted to determine whether or how the effects of masks on face perception have changed over time as individuals adapted to the effects of masks. Consequently, the first aim of the current study was to examine the effects of masks and other facial occlusions (sunglasses) on identification and social judgements at three different time points, spanning more than 13 months.

### Variability of effects across individuals

The second aim of the current study was to examine how the effects of masks on identification vary between individuals. It is apparent from data collected in previous studies that the effects of masks in identification vary substantially between individuals (Marini et al., 2021; Noyes et al., 2021). However, it is unclear what factors can account for the variability in the effects of masks on face processing.

One possibility is that the effects of masks on identification might be associated with face recognition ability. Face recognition varies substantially in the general population (Bowles et al., 2009; Germine et al., 2011). At one extreme of this variability, there are some individuals who have very poor face recognition skills, despite relatively normal intellectual capacities and low-level vision—this is referred to as developmental prosopagnosia (DP; also sometimes referred to as ‘congenital prosopagnosia’; Bate & Tree, 2017; Corrow et al., 2016; Susilo & Duchaine, 2013). At the other extreme, some individuals have extraordinarily good face recognition skills—these individuals have been referred to as “super-recognisers” (Bate et al., 2018; Bennetts et al., 2017; Bobak et al., 2016a; Ramon, 2021; Russell et al., 2009).

Currently, we do not know of any research in DP that has investigated naturalistic face occlusions such as sunglasses and face coverings. While face perception in DP is heterogeneous (Bate et al., 2019c; Dalrymple et al., 2014; Klargaard et al., 2018; Palermo et al., 2011), there is some evidence that, on a group level, individuals with DP might show particular difficulty with naturalistic face transformations (e.g., matching images despite changes in viewpoint, lighting, or other transformations) (White et al., 2017). Further, some individuals with DP also report relying on unusual feature-based or extra-facial strategies to recognise individuals (Adams et al., 2020; Murray et al., 2018). The use of atypical strategies in DP is supported by eye-tracking research which shows that, compared to typical controls, some individuals with DP spend a higher proportion of their time looking at unusual areas of the face (e.g., the mouth, Bobak et al., 2017; hairline, neck, and chin, Schwarzer et al., 2007) or body (Bobak et al., 2017). This may make individuals with DP particularly vulnerable to the effects of masks, particularly in cases where extra-facial information is limited or unreliable.

While SRs tend to perform exceptionally well on tasks involving face memory, their performance on face perception tasks is also heterogeneous (e.g., Bate et al., 2018, 2019d; Bobak et al., 2016a; Noyes et al., 2021). Furthermore, SRs are not impervious to the same biases that affect face recognition in the typical population—for example, SRs display “own-age” and “own-ethnicity” biases (Bate et al., 2019a, 2020). Noyes et al. (2021) also found that SRs performed worse when matching unfamiliar faces with masks or sunglasses, compared to uncovered faces. Notably, though, their pattern of performance across conditions was different to that of typical perceivers: while SRs were equally good at matching faces with sunglasses and face coverings, typical perceivers showed slightly better performance for faces with sunglasses than those with masks. As for typical perceivers, it is unclear how SRs’ performance might be affected by multiple occlusions (sunglasses and masks).

### The current study

This study used edited stimuli from a pre-existing, well-validated database of face images to examine the effects of masks and sunglasses (alone and in combination) on face identity matching and social judgements. To determine the effects over time, data was collected from different groups of participants with typical face recognition abilities at three points in time between June 2020 and July 2021 (Experiment 1). To examine whether face recognition abilities are associated with the effects of different face occlusions, we compared performance on the matching task across three groups of individuals: those with typical face recognition, individuals with DP, and SRs; and examined the relationship between self-reported face recognition ability and the effects of masks and sunglasses (Experiment 2).

## Experiment 1

### Methods

#### Participants

Data for Experiment 1 was collected at three different timepoints: June 2020, February 2021, and August 2021. A total of 150 participants (50 per time point) completed the study. Participants were recruited via online participant recruitment services (Prolific.ac and Testable Minds), with the restriction that the study should be available to people who were living within the UK. Subsequently, 15 participants were excluded from analysis: nine were outside the age range for the study (18–60 years of age), and six were outliers in the face matching task (> 3 SDs from the mean in measures of sensitivity or bias). One participant only completed the ratings tasks, not the matching task.

The final sample for analysis included 135 participants (62 female, 71 male, 2 other, *M*_age_ = 32.61 years, SD = 10.88); 44 in June 2020 (26 female, 18 male, *M*_age_ = 33.64 years, SD = 10.88); 44 in February 2021 (18 female, 26 male, *M*_age_ = 28.75 years, SD = 7.43); and 47 in August 2021 (18 female, 27 male, 2 other, *M*_age_ = 35.26 years, SD = 10.57). Power calculations (G*Power 3.1.9.2) indicated that this sample size was sufficient to detect an effect of masks and an interaction between masks and timepoint with a small-to-medium effect size (*d* = 0.24) with 90% power, assuming a correlation of *r* = 0.68 between the within-subjects variables (this was based on the data obtained in Experiment 1). Noyes et al. (2021), reported an effect size of *d* = 0.57 for unpractised control participants; thus, our study has sufficient power to detect smaller effects of masks than have previously been found in the literature.

The vast majority of the sample (111) identified their ethnicity as Caucasian/White (44 from June 2020; 27 from February 2021; 38 from August 2021); 19 identified their ethnicity as Asian or Pacific Islander (0 from June 2020; 14 from February 2021; five from August 2021); three identified their ethnicity as Black (0 from June 2020; one from February 2021; two from August 2021, one identified their ethnicity as Hispanic or Latino (from February 2021), and two identified their ethnicity as Other (both from August 2021).

#### Materials

##### Face images

The target stimuli consisted of 60 identities (30 female) from the Glasgow Unfamiliar Face Database (GUFD; Burton et al., 2010). Sixty identities (30 female), matched to the target stimuli in gender and similar in age, skin tone, hair colour, and hair style, were selected as distractor images (as in the Glasgow Face Matching Test, there was some overlap between test and distractor identities to ensure a good match between images). The GUFD images have been used for prior research and include images of the same face, with a neutral facial expression, captured with multiple cameras. The stimuli thus include some small variations in face size, colouration, lighting, head angle, and hairstyle, making it difficult to match images based on pictorial cues (e.g., skin tone, specific idiosyncrasies in an image) alone.

**Fig. 1:**
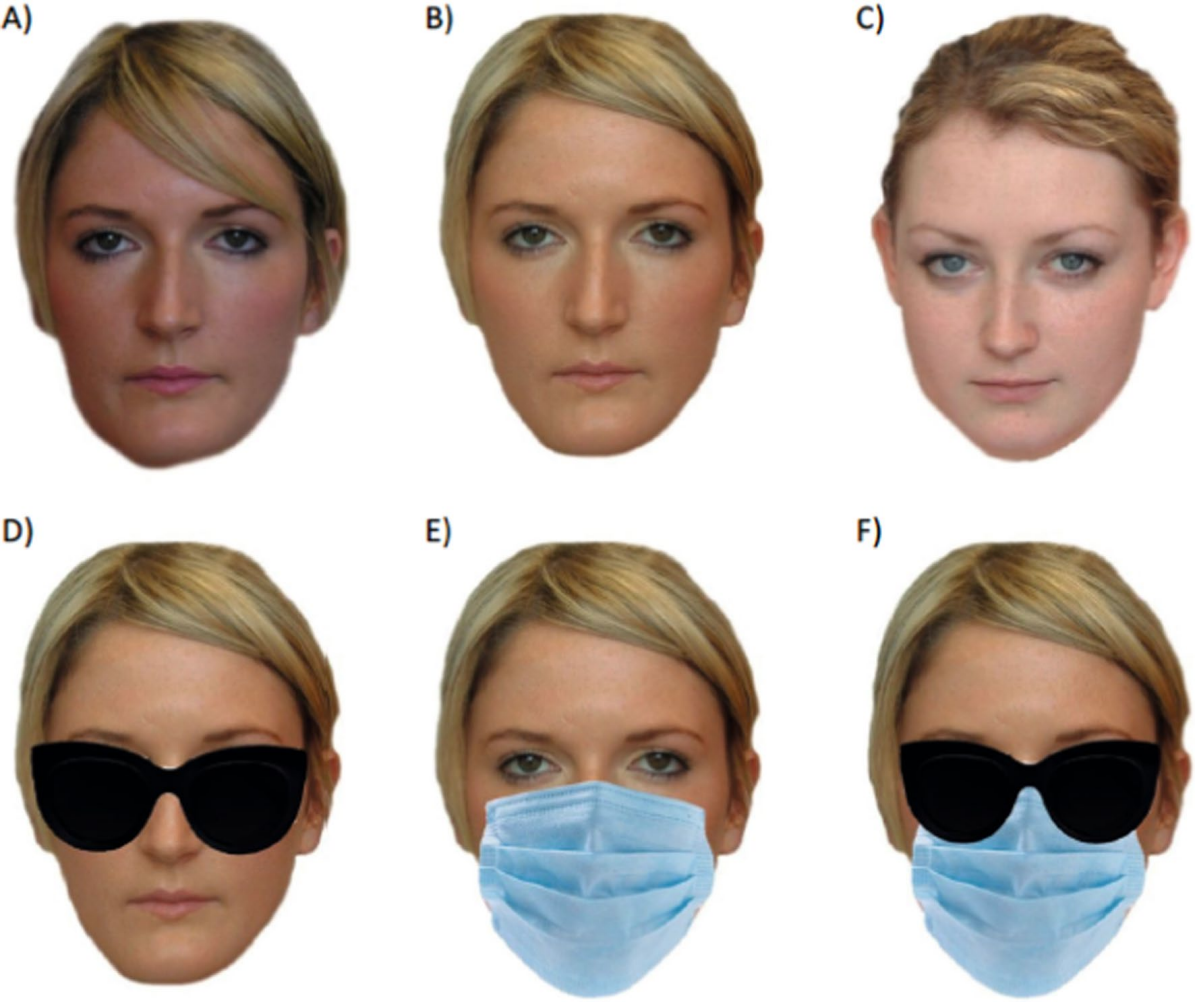
Examples of stimuli used in the matching and ratings tasks. **A** An unedited comparison image; **B** An unedited target image; **C** An unedited distractor image; **D** Sunglasses only image; **E** Mask only image; **F** Sunglasses and mask image

Two images taken with different cameras (C1 and C2) were selected for each target identity. All images were selected to show the face from a roughly frontal viewpoint (with variation of up to 10 degrees), but the pairs included some variation in colouration, hairstyles, and face size. Some pairs also showed small changes in eye gaze or facial pose (e.g., mouth closed/mouth slightly open). Images were resized to 800 × 600 pixels. The first image (C1) was not edited further. The second image of each individual (C2) was edited in Adobe Photoshop to show the face wearing (1) a medical-style face mask; (2) sunglasses; and (3) both sunglasses and a face mask (Fig. 1). Images of sunglasses and face masks were selected via an online search (Google images). To prevent participants becoming overly familiar with the accessories, multiple versions of sunglasses and masks were selected (3 masks, 6 sunglasses) and applied to equal numbers of faces (within a single identity, the same accessories were always shown). While the accessories varied in terms of colour and style, the basic shape of the accessories was consistent within genders (male sunglasses were a slightly different shape to female sunglasses), and the images were resized and warped to ensure they covered a similar area of each face. A single image of each distractor identity (from the C2 camera) was selected and edited in the same way as the target images.

In sum, for each unedited target face, there were four “matching” images of the same identity and four “mismatching” images of a different identity (unedited, mask only, sunglasses only, mask and sunglasses). For clarity, the unedited image from camera 1 will be referred to as the “comparison image”, the matching images from camera 2 as “target images”, and the mismatching images as “distractor images”.

#### Design and procedure

Participants completed four face processing tasks. The first three tasks involved rating the attractiveness, competence, and trustworthiness of each face. The final task involved matching faces based on identity. The design for all tasks was similar: fully within-subjects, with all participants providing ratings/accuracy data for faces in all four conditions (unedited/control, mask only, sunglasses only, sunglasses and mask). Timepoint was also included as a between-subjects variable in the analyses.

For each rating task participants were asked to rate 60 target images (15 in each condition: unedited/control, mask only, sunglasses only, sunglasses and mask) for trustworthiness, competence, and attractiveness on 7-point Likert scales. Participants viewed the target face in the centre of the screen, with the Likert scale presented above it. The Likert scales ranged from 1 (Very Untrustworthy/Unattractive/Incompetent) to 7 (Very Trustworthy/Attractive/Competent), with 4 representing “Neutral”. Responses were made via keypress (the 1–7 keys on the keyboard). There was no time limit to respond, and images stayed on screen until a response was recorded. Prior to each task, there were three practice trials. The ratings tasks were blocked (so participants provided all the trustworthiness ratings in a single block, all the competence ratings in a separate block etc.), and their order of presentation was randomised between participants. The order of presentation of faces in each ratings task was randomised. The allocation of different faces to different conditions was counterbalanced between participants.

For the matching task participants saw pairs of images (one comparison image, paired with either a target or distractor image) presented simultaneously, and were asked to indicate whether the two images depicted the same person or two different people by clicking the “SAME” or “DIFFERENT” button onscreen. There was no time limit to respond, and images stayed on screen until a response was recorded. Participants completed 120 trials in total: 30 in each condition (unedited/control, mask only, sunglasses only, sunglasses and mask), of which half were same identity trials and half were different identity trials.

There were two practice trials at the beginning of the task, and a short break in the middle of the task. Participants did not receive feedback on the practice trials. As in the ratings task, the allocation of different faces to different conditions was counterbalanced across participants, and trials were presented in a random order. Following the matching task, participants completed the PI20 (see Experiment 2 for further details).

Participants at the second and third timepoints (February and August 2021) viewed the same identities in the same conditions (i.e., the same faces were presented with sunglasses/masks/both) in the ratings and matching tasks. The target images presented in the matching task were the same as the images used in the ratings task, meaning that each target image was viewed four times across the entire experiment. Due to a difference in task programming, we were unable to control whether participants at the first timepoint viewed the same faces in the same conditions for the ratings and matching tasks. Participants were not informed that they would see the same identities in the rating and matching tasks.

All data collection took place online via platforms designed for online tasks (Testable.org and Qualtrics). Prior to the experiment, all participants provided informed consent via an online consent form. This project was approved by the institutional Research Ethics Committee, references 11697-A-Apr/2020- 25416-1; 11697-A-Jul/2021- 33456-1; 21052-A-Jul/2021- 33366-2.

#### Statistical analyses

##### Matching task

Scores for all participants were calculated in terms of hits (the number of correct “same” responses) and correct rejections (the number of correct “different” responses). This data was also used to calculate signal detection theory (SDT) measures of sensitivity. Due to the non-normal distribution of the data (many participants achieved perfect or near-perfect accuracy in some conditions), the analysis for this task used non-parametric measures of sensitivity (*A*) and bias (*b*) (Zhang & Mueller, 2005). The measure *A* ranges from 0 (chance performance) to 1 (perfect performance); the measure *b* is used as an indicator of response bias (i.e., whether the participant has a tendency to say that the images are the same or different). A *b* of 1 indicates a neutral response criterion, whereas a higher score indicates conservative responding (a tendency to indicate that a two faces were different) and a lower score indicates more liberal responding (a tendency to indicate that the two faces were the same) (Macmillan & Creelman, 2005). Examination of the average *A* and *b* across conditions revealed six participants who were extreme outliers (> 3 SDs from the mean) on at least one measure; these participants were excluded from all analyses.

We analysed response time (RT) to trials with correct responses only. Any RTs greater than 3SD from a participants’ mean RT were excluded from calculations.

##### Ratings tasks

The mean rating for each condition was calculated for each participant for the three ratings tasks. Mean ratings could range from 1 to 7.

All the data from the study can be accessed at https://osf.io/m2ch8/?view_only=a5b8edc88bcc4d6ca3f7b9b56b57b0d6.

##### Preliminary analyses

Previous research suggests that age can influence face identity perception (Bowles et al., 2009; Megreya & Bindemann, 2015) and some social judgements (Zebrowitz et al., 2013). Consequently, participants were divided into two age groups: younger adults (18–39 years old) and older adults (40–59 years old), and data from all tasks was entered into a series of ANOVAs including age group as a between-subjects factor. The main effects of age group and interactions with other variables were not significant for any of the key dependent variables (*A*, ratings), all *p*’s > .100. Furthermore, entering age as a covariate in the analyses did not change the pattern of results. Consequently, age was excluded from further analyses.

Initial examination of the data revealed departures from normality (Shapiro–Wilk *p*’s < .05) in many variables, with the data (particularly from the matching task) showing substantial skew. However, as ANOVA models are relatively robust to departures from normality (Blanca et al., 2017), and there was no evidence of violation of the homogeneity of variance assumption (all Levene’s *p*’s > .05) we proceeded with the planned analyses.

### Results

#### Matching task

**Fig. 2:**
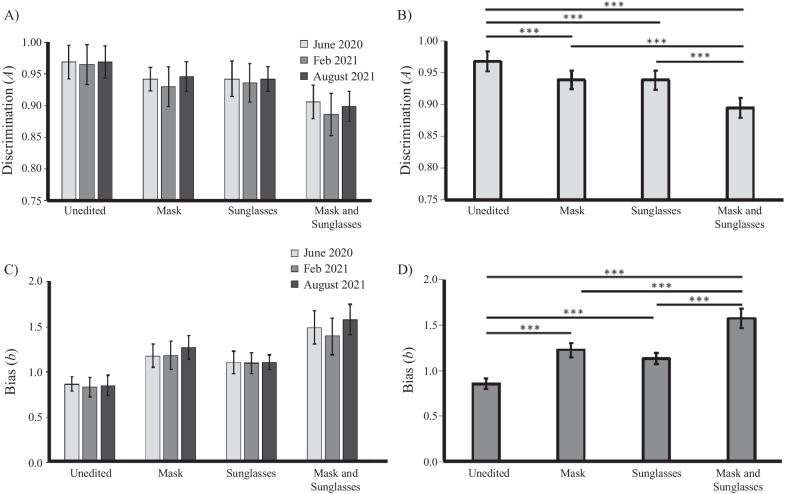
Signal detection theory measures from the matching task. **A**, **B** show mean discrimination (**A**); **C**, **D** show mean bias (b) for each image condition. **A**, **C** show data separated across timepoints; **B**, **D** show data averaged across timepoint. Error bars represent 95% within subjects confidence intervals, calculated according to the Cosineau-Morey method (as reported in Baguley, 2012). In **B**, **D**, lines with *** indicate that Bonferroni-corrected pairwise comparisons between conditions were significant, *p* < .05

Performance on the matching task at each timepoint is displayed in Table 1. Performance is displayed in Figs. 2 (SDT measures) and 3 (accuracy and response time). SDT data from the matching task (*A* and *b*) was initially entered into two 3 (timepoint: June 2020; February 2021; August 2021) × 2 (mask: mask; no mask) × 2 (sunglasses: sunglasses; no sunglasses) ANOVAs.

**Table 1:** Mean (SD) for each timepoint for the matching and ratings tasks

Measure	Unedited faces			Mask only			Sunglasses only			Mask and sunglasses		
	Jun 2020	Feb 2021	Aug 2021	Jun 2020	Feb 2021	Aug 2021	Jun 2020	Feb 2021	Aug 2021	Jun 2020	Feb 2021	Aug 2021
SDT measures												
*A* (sensitivity)	0.97 (0.04)	0.96 (0.04)	0.97 (0.04)	0.94 (0.05)	0.93 (0.06)	0.95 (0.05)	0.94 (0.06)	0.94 (0.06)	0.94 (0.06)	0.91 (0.07)	0.89 (0.08)	0.90 (0.07)
*B* (bias)	0.87 (0.24)	0.84 (0.24)	0.85 (0.22)	1.18 (0.52)	1.18 (0.58)	1.27 (0.55)	1.10 (0.48)	1.10 (0.46)	1.11 (0.45)	1.49 (0.71)	1.40 (0.76)	1.58 (0.77)
Accuracy												
Matched identity trials	0.98 (0.03)	0.98 (0.04)	0.98 (0.04)	0.89 (0.11)	0.88 (0.11)	0.88 (0.12)	0.90 (0.11)	0.89 (0.10)	0.90 (0.12)	0.79 (0.15)	0.77 (0.18)	0.76 (0.17)
Mismatched identity trials	0.90 (0.14)	0.89 (0.14)	0.91 (0.15)	0.92 (0.11)	0.88 (0.15)	0.93 (0.11)	0.90 (0.15)	0.89 (0.15)	0.91 (0.12)	0.90 (0.14)	0.88 (0.12)	0.90 (0.14)
Response time (correct responses)												
Matched identity trials	2530 (2374)	1865 (676)	1914 (976)	2896 (2993)	2445 (1064)	2766 (2006)	2988 (2280)	2548 (1482)	2657 (1380)	3380 (2967)	3316 (2191)	3298 (2141)
Mismatched identity trials	2690 (2620)	2544 (2463)	2405 (1227)	2517 (1432)	2495 (1169)	2414 (1613)	2679 (1536)	2764 (1701)	2598 (1316)	2711 (1757)	2766 (1340)	2908 (2201)
Mean ratings												
Trustworthiness	4.10 (0.86)	4.40 (0.85)	4.22 (0.68)	4.28 (0.68)	4.55 (0.75)	4.14 (0.90)	3.32 (1.06)	3.35 (0.85)	3.49 (0.84)	3.04 (1.02)	3.38 (1.21)	3.26 (0.99)
Competence	4.22 (1.04)	4.39 (0.83)	4.36 (0.71)	4.57 (0.66)	4.72 (0.91)	4.44 (0.86)	3.55 (1.06)	3.81 (1.00)	3.74 (0.91)	3.56 (1.16)	3.77 (1.12)	3.77 (0.90)
Attractiveness	3.57 (0.88)	3.70 (0.91)	3.14 (0.92)	3.65 (0.80)	3.81 (0.72)	3.41 (0.89)	3.18 (0.94)	3.35 (0.79)	3.01 (0.93)	2.94 (1.01)	3.16 (0.92)	2.97 (0.96)

The ANOVA on *A* revealed main effects of mask, *F*(1,131) = 102.08, *p* < .001, *ηρ*^2^ = .44, and sunglasses, *F*(1,131) = 68.38, *p* < .001, *ηρ*^2^ = .34. On average, unmasked faces (*M* = 0.95, SD = 0.04) were matched better than masked faces (*M* = 0.92, SD = 0.04); and faces without sunglasses (*M* = 0.95, SD = 0.05) were matched better than faces with sunglasses (*M* = 0.92, SD = 0.05).

These main effects were superseded by an interaction between masks and sunglasses, *F*(1,131) = 5.04, *p* = .026, *ηρ*^2^ = .04 (see Fig. 2). Simple pairwise comparisons (Bonferroni-corrected) confirmed that masks or sunglasses alone impaired recognition compared to the unedited faces, *p*’s < .001, and faces with both masks and sunglasses were matched significantly worse than sunglasses or masks alone, *p*’s < .001. There was no significant difference between performance with masks alone and performance with sunglasses alone, *p* > .99. However, the effect of masks on identification (i.e., the difference between performance for masked and unmasked faces) was slightly, but significantly, higher when the faces were depicted with sunglasses (*M* = 0.043, SD = 0.07) than without sunglasses, (*M* = 0.028, SD = 0.04), *F*(1,131) = 5.04, *p* = .026, *ηρ*^2^ = .04.

There was no main effect of timepoint, *F*(2,131) = 0.70, *p* = .500, *ηρ*^2^ = .01, and timepoint did not interact significantly with any other effects, mask × timepoint: *F*(2,131) = 1.06, *p* = .351, *ηρ*^2^ = .02, sunglasses × timepoint: *F*(2,131) = 0.19, *p* = .825,1 *ηρ*^2^ = .00, mask × sunglasses × timepoint: *F*(2,131) = 0.24, *p* = .790, *ηρ*^2^ = .00. Thus, the effects of masks and sunglasses, alone or in combination, did not significantly differ across timepoints.

The ANOVA on *b* revealed significant main effects of masks, *F*(1,131) = 111.44, *p* < .001, *ηρ*^2^ = .46, and sunglasses, *F*(1,131) = 38.72, *p* < .001, *ηρ*^2^ = .23. Masked faces (*M* = 1.35, SD = 0.41) led to more conservative patterns of responding than unmasked faces (*M* = 0.98, SD = 0.41); likewise, faces with sunglasses (*M* = 1.30, SD = 0.43) led to more conservative responding than faces without sunglasses, (*M* = 1.03, SD = 0.43) (see Fig. 2).

There was no main effect of timepoint on bias, *F*(2,131) = 0.50, *p* = .607, *ηρ*^2^ = .01, and no interactions were significant, all *p*’s > .30.

##### Effects of trial type

To explore the effects of masks on matched and mismatched trials separately, accuracy data was entered into a 3 (timepoint: June 2020; February 2021; August 2021) × 2 (mask: mask; no mask) × 2 (sunglasses: sunglasses; no sunglasses) × 2 (trial type: matched; mismatched) ANOVA. As in the *A* analysis, the main effects of mask and sunglasses, and the interaction between masks and sunglasses, were significant, mask: *F*(1,131) = 118.86, *p* < .001, *ηρ*^2^ = .48; sunglasses, *F*(1,131) = 75.71, *p* < .001, *ηρ*^2^ = .37, mask × sunglasses: *F*(1,131) = 4.38, *p* = .038, *ηρ*^2^ = .02. Pairwise comparisons (Bonferroni-corrected) on the interaction showed a similar pattern to the *A* data, with unedited faces matched significantly more accurately than faces with masks or sunglasses, *p*’s < .001, and faces with a single occlusion matched better than faces with both masks and sunglasses, *p*’s < .001, but no significant difference between faces with masks only and faces with sunglasses only, *p* = 1.

**Fig. 3:**
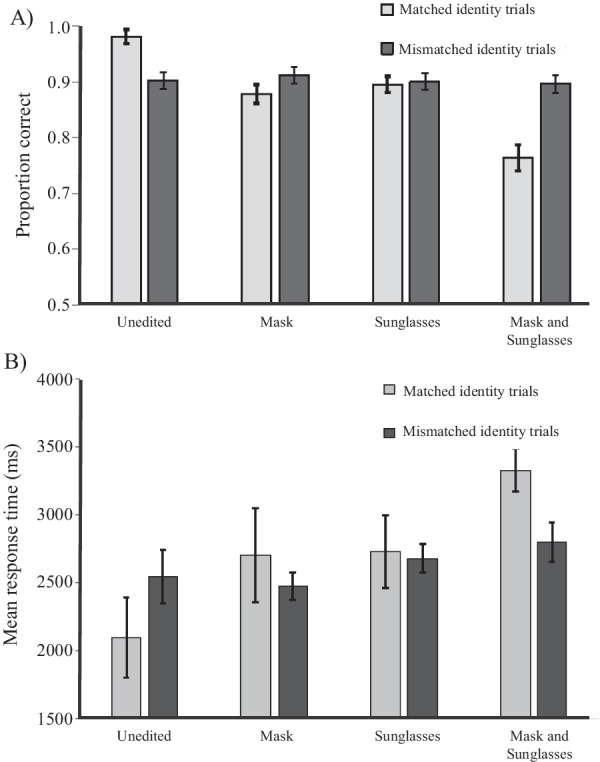
**A** Mean accuracy and **B** mean response time (correct trials only) in matched identity (“Same”) and mismatched identity (“Different”) trials. Error bars represent 95% within subjects confidence intervals, calculated according to the Cosineau-Morey method (as reported in Baguley, 2012)

The main effect of trial type was not significant, *F*(1,131) = 1.63, *p* = .203, *ηρ*^2^ = .01, but trial type interacted with both mask, *F*(1,131) = 101.90, *p* < .001, *ηρ*^2^ = .44, and sunglasses, *F*(1,131) = 32.93, *p* < .001, *ηρ*^2^ = .20. The three-way interaction was not significant, *F*(1,131) = 0.31, *p* = .578, *ηρ*^2^ = .00. Follow-up comparisons showed that neither masks nor sunglasses made a significant difference to accuracy in mismatched trials, *p*’s = 1, however, accuracy in matched identity trials significantly decreased when either masks or sunglasses were introduced, *p*’s < .001 (see Fig. 3).

Once again, the effects of masks and sunglasses did not differ across timepoints: the main effect of timepoint was not significant, *F*(2,131) = 0.54, *p* = .587, *ηρ*^2^ = .01; and none of the interactions involving timepoint were significant, all *p*’s > 0.5.

##### Response time

A 3 (timepoint: June 2020; February 2021; August 2021) × 2 (mask: mask; no mask) × 2 (sunglasses: sunglasses; no sunglasses) × 2 (trial type: matched; mismatched) ANOVA revealed a similar pattern of findings to the main analysis on accuracy for matched and mismatched trials: there were significant main effects of masks and sunglasses, but not trial type, mask: *F*(1,131) = 26.85, *p* < .001, *ηρ*^2^ = .17; sunglasses, *F*(1,131) = 65.49, *p* < .001, *ηρ*^2^ = .33, trial type: *F*(1,131) = 1.65, *p* = .201, *ηρ*^2^ = .01. There were significant interactions between mask and trial type, *F*(1,131) = 27.25, *p* < .001, *ηρ*^2^ = .17, and sunglasses and trial type: *F*(1,131) = 13.18, *p* < .001, *ηρ*^2^ = .09. Both masks and sunglasses led to slower responses than unoccluded faces in matched identity trials, *p*’s < .001. Response times to masked and unmasked faces were not significantly different for mismatched trials, *p* > 0.9, but responses to faces with sunglasses were slower than to faces without sunglasses in mismatched identity trials, *p* = .02.

There were no significant main effects of interactions with timepoint, *p*’s > 0.08, and no other interactions were significant, *p*’s > 0.1.

##### Difficult trials

Participants performed very well in the matching task overall (see Figs. 2 and 3). It is possible that ceiling effects could obscure some subtle differences between conditions or timepoints; consequently, we repeated the analyses on a reduced dataset that contained a subset of more difficult trials. Due to counterbalancing, there were four sets of faces (each with 15 target identities) presented to participants. We selected the five target identities with the highest baseline accuracy in each set (based on unedited trials) from the analysis. Data for these faces was removed from all conditions. This resulted in a reduced dataset, containing the most difficult 2/3 of trials in the experiment. Average accuracy in the unedited condition for the reduced dataset was 92.4% (compared to 94.1% in the full dataset); this is similar to the levels of accuracy reported for the original Glasgow Unfamiliar Faces Test (89.9% in Burton et al., 2010). The analyses reported above were repeated on this more difficult dataset. For brevity, we have only reported a brief summary of the analyses here; however, the data from the more difficult trials is openly available alongside the full dataset at https://osf.io/m2ch8/?view_only=a5b8edc88bcc4d6ca3f7b9b56b57b0d6.

Overall, the pattern of results for the reduced dataset was identical to the full dataset. For sensitivity (*A*), the ANOVA revealed no significant effects of timepoint, all *p*’s > 0.2, but all main effects and interactions involving masks and sunglasses were significant, *p*’s < .05. As in the main analysis, participants performed better with unedited faces than those with masks or sunglasses, *p*’s < .001; and worse with masks and sunglasses compared to masks or sunglasses in isolation, *p*’s < .02. There was no significant difference in performance for faces shown with sunglasses or with masks alone, *p* > 0.9. Similarly, for accuracy, the results for the reduced dataset mirrored those for the full dataset: no significant main effect or interactions involving timepoint, *p*’s > 0.3, and the same pattern of performance across conditions and trial types as in the complete dataset.

#### Ratings tasks

The mean ratings given to faces in each condition at each timepoint are shown in Table 1. Separate 3 (timepoint: June 2020; February 2021; August 2021) × 2 (mask: mask; no mask) × 2 (sunglasses: sunglasses; no sunglasses) ANOVAs were carried out on the trustworthiness, competence, and attractiveness ratings.

##### Trustworthiness

**Fig. 4:**
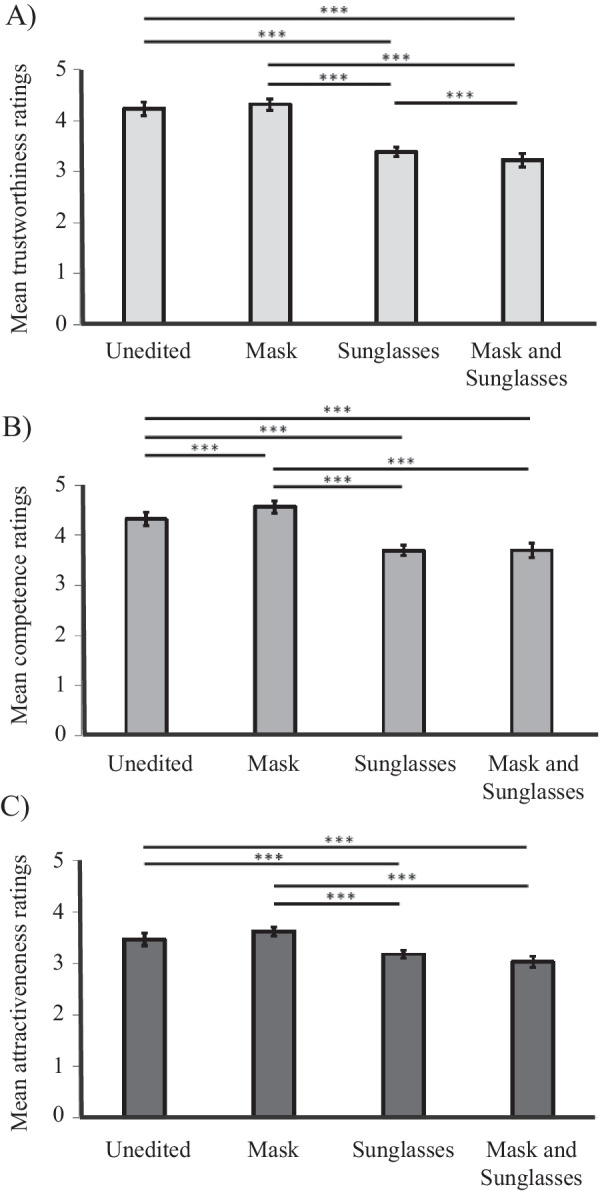
Mean **A** trustworthiness; **B** competence; and **C** attractiveness ratings for each image condition, averaged across timepoint. Error bars represent 95% within subjects confidence intervals, calculated according to the Cosineau-Morey method (as reported in Baguley, 2012). Lines with ** indicate that Bonferroni-corrected pairwise comparisons between conditions were significant, *p* < .05

The main effect of masks on trustworthiness judgements was not significant, *F*(1,132) = 0.34, *p* = .558, *ηρ*^2^ = .01. The main effect of sunglasses on trustworthiness judgements was significant, *F*(1,132) = 163.97, *p* < .001, *ηρ*^2^ = .55; faces with sunglasses (*M* = 3.31, SD = 0.80) were rated as less trustworthy, on average, than those without sunglasses (*M* = 4.28, SD = 0.80). There was also a significant interaction between masks and sunglasses, *F*(1,132) = 12.48, *p* < .001, *ηρ*^2^ = .06. Follow-up simple main effects analyses revealed that there was a significant negative effect of masks on trustworthiness judgements when the images were shown wearing sunglasses, *p* = .021, but not when the images were shown without sunglasses, *p* = .27 (see Fig. 4).

The main effect of timepoint was not significant, *F*(2,132) = 1.42, *p* = .247, *ηρ*^2^ = .02, nor were the two-way interactions between timepoint and masks or sunglasses, *p*’s > 0.20, or the three-way interaction between masks, sunglasses, and timepoint, *F*(2,132) = 2.33, *p* = .10, *ηρ*^2^ = .03.

##### Competence

Similar to trustworthiness, the analysis on competence judgements revealed no main effect of masks, *F*(1,132) = 3.67, *p* = .058, *ηρ*^2^ = .03, but a significant main effect of sunglasses, *F*(1,132) = 88.26, *p* < .001, *ηρ*^2^ = .40 and a significant interaction between masks and sunglasses, *F*(1,132) = 14.15, *p* < .001, *ηρ*^2^ = .10. While the main effect of sunglasses was similar to trustworthiness, with sunglasses leading to lower competence ratings on average (sunglasses: *M* = 3.70, SD = 0.82, no sunglasses: *M* = 4.45, SD = 0.82), the pattern of ratings for the mask × sunglasses interaction diverged substantially from trustworthiness judgements. When faces were depicted with sunglasses, masks did not have a significant effect on competence ratings, *p* = 1; however, when faces were depicted without sunglasses, masks increased competence ratings, *p* = .006 (see Fig. 4).

There was no main effect of timepoint, *F*(2,132) = 0.95, *p* = .388, *ηρ*^2^ = .01, and no interactions with timepoint were significant, *p*’s > 0.08.

##### Attractiveness

As for both trustworthiness and competence, the ANOVA on attractiveness judgements revealed no significant main effect of masks, *F*(1,132) = 0.03, *p* = .955, *ηρ*^2^ = .00, but a significant main effect of sunglasses, *F*(1,132) = 68.88, *p* < .001, *ηρ*^2^ = .34, and a significant interaction between masks and sunglasses, *F*(1,132) = 22.95, *p* < .001, *ηρ*^2^ = .15. Once again, sunglasses led to lower ratings on average (sunglasses: *M* = 3.10, SD = 0.81, no sunglasses: *M* = 3.54, SD = 0.81). Pairwise comparisons did not reveal a difference between unedited and masked faces, *p* = .135, or faces depicted with sunglasses or sunglasses and masks, *p* = .104. However, follow-up analysis exploring the interaction revealed that the effect of masks on attractiveness ratings (i.e., the difference between ratings for masked and unmasked faces) was significantly different for faces depicted with (*M* = 0.15, SD = 0.76) and without sunglasses (*M* = −0.15, SD = 0.76), *F*(1,132) = 22.95, *p* < .001, *ηρ*^2^ = .15 (see Fig. 4).

Once again, there was no significant effect of timepoint, *F*(1,132) = 2.83, *p* = .062, *ηρ*^2^ = .04, and no interaction between timepoint and any other variable, *p*’s > 0.1.

### Discussion

Experiment 1 examined the effects of different facial coverings (masks, sunglasses) on face perception across three timepoints. Consistent with previous research (Carragher & Hancock, 2020; Graham & Ritchie, 2019; Noyes et al., 2021), the findings suggest that face masks and sunglasses have a significant effect on face matching ability: both result in a significant decrease in sensitivity in a face matching task. The effects of masks on trait judgements were somewhat variable, and depended on the judgement being made and the presence of other face coverings (i.e., sunglasses).

In general, the effects of masks were consistent across the three timepoints measured in this research. This suggests that extended exposure to mask-wearing over the course of a year has not reduced the effects of masks on face processing. However, while the effects of face coverings did not differ significantly over time, there was substantial variability between participants, regardless of timepoint. For example, within our sample, the mask effect varied from negligible or even negative (a mask advantage) to a 23% reduction in overall face matching accuracy for some participants. On a broader level, the variance associated with individual differences in the *A* analysis equated to 51.5% of the total variance in the data. In Experiment 2, we examined whether this variability was related to individual differences in face recognition ability. First, we compared the effects of masks and sunglasses on face matching in individuals with extremely good (super-recognisers) and very poor (developmental prosopagnosia) face recognition. Second, we examined whether the effects of masks correlate with self-reported face recognition ability in the general population.

## Experiment 2

### Methods

#### Participants

Twelve individuals with DP (11 female, 1 male, *M*_age_ = 47.00 years, SD = 10.08) and 10 SRs (4 female, 6 male, *M*_age_ = 40.60 years, SD = 10.53) took part in Experiment 2. All DP and SR participants identified their ethnicity as Caucasian/White. All had contacted our lab following media coverage of prosopagnosia and super recognition research, and had completed a series of screening tasks to confirm their face recognition abilities.

Individuals with suspected DP reported severe difficulties with everyday face recognition. Following standard diagnostic protocols used by many labs in the field (Barton et al., 2019; Bate et al., 2019c; Corrow et al., 2016; Dalrymple & Palermo, 2016), all individuals with DP performed significantly below published age-matched control cut-offs on two tests of face recognition: the Cambridge Face Memory Test (CFMT; Duchaine & Nakayama, 2006; for cut-offs see Bowles et al., 2009) and a famous faces test (Bate et al., 2019b). Participants with DP also completed the Cambridge Face Perception Test (CFPT; Duchaine et al., 2007). Because face perception can sometimes be preserved in DP, CFPT scores are typically not used as an absolute diagnostic criterion (Bate & Tree, 2017; Dalrymple & Palermo, 2016), and are not regarded as such in the current study. However, given the perceptual nature of the tasks, they are provided for all individuals with DP in the current study. No individual reported a history of socio-emotional, psychiatric or neurological disorder. Data from the CFMT, famous faces task, and CFPT for each participant with DP is presented in Table 2.

**Table 2:** Age and gender for each individual DP participant, together with standardized scores for performance on the CFMT (Duchaine & Nakayama, 2006), CFPT (Duchaine et al., 2007), and a famous faces test that was created within our laboratory (Bate et al. 2019b)

	Age	Gender	CFMT	CFPT	Famous Faces
DP01	33	F	− 2.26	1.25	− 4.58
DP02	36	F	− 3.15	0.93	− 6.89
DP03	53	F	− 2.90	2.40	− 9.56
DP04	49	F	− 3.27	2.40	− 5.89
DP05	53	F	− 3.27	3.55	− 7.78
DP06	54	F	− 2.01	2.40	− 9.26
DP07	58	F	− 3.15	1.42	− 4.62
DP08	56	F	− 2.39	1.91	− 3.58
DP09	45	F	− 2.77	1.58	− 3.95
DP10	52	F	− 2.90	2.73	− 8.73
DP11	49	F	− 2.01	2.40	− 3.30
DP12	26	F	− 3.40	0.93	− 6.22

Control scores are taken from the relevant publications. Note CFPT scores are reversed (scores equate to errors), so a positive z-score indicates poorer performance than controls

Individuals who believed they were SRs completed a series of challenging face recognition tasks, designed to identify individuals with extremely good face recognition. All SRs included in the current research had obtained scores more than 1.96 SDs above control norms (norms were taken from Bate et al., 2018) on the extended form of the Cambridge Face Memory Test (CFMT + : Russell et al., 2009) and the Models Memory Test (MMT: Bate et al., 2018). The CFMT + is a dominant test of face memory that is frequently used for SR identification (e.g. Bennetts et al., 2017; Bobak, Pampoulov & Bate, 2016; Phillips et al., 2018); the MMT is a newer test of face memory that adopts the CFMT + paradigm (see Bate et al., 2018). SR participants also completed the Pairs Matching Test (PMT: Bate et al., 2018), a challenging test of face matching ability. Similar to DP, face matching abilities can vary in SR (Bate et al., 2018, 2019d; Bobak et al., 2016b; Davis et al., 2016), and performance on the PMT was not used as an inclusion criterion in the current study. Data from the CFMT + , MMT, and PMT for each SR participant is provided in Table 3.

**Table 3:** Age and gender for each individual SR participant, together with standardized scores for performance on the CFMT + (Russell et al., 2009), MMT, and PMT (Bate et al., 2018)

	Age	Gender	CFMT +	MMT	PMT
SR01	24	M	2.84	2.46	2.86
SR02	39	M	2.64	2.06	1.42
SR03	54	M	2.74	2.46	2.86
SR04	37	M	2.84	3.26	2.28
SR05	46	F	2.64	2.86	2.00
SR06	43	M	2.44	3.10	2.00
SR07	58	M	2.94	1.98	0.56
SR08	39	F	2.04	2.46	3.14
SR09	39	F	2.54	2.06	1.13
SR10	27	F	3.14	3.34	2.57

Control scores are taken from the relevant publications

Given the difference in age between the two groups, we created separate control samples for the DP and SR participants.^2^ The control samples for Experiment 2 consisted of a subset of participants who completed Experiment 1. Each subset was selected to be broadly similar in age (mean and distribution) to the DP and SR groups. Data for the DP and SR groups was collected at a similar time to the final timepoint of control data (late July–August 2021). Consequently, the control data was drawn from participants who completed the tasks at the final timepoint (August 2021).

All participants in Experiment 1 completed the PI20, (Shah et al., 2015a) a 20-item self-report measure of face recognition ability. Perceived face processing ability is assessed via questions such as “I often mistake people I have met before for strangers”, “Without hearing people's voices, I struggle to recognize them”, “My friends and family think I have bad face recognition or bad face memory”, and “It is easy for me to recognize individuals in situations that require people to wear similar clothes (e.g. suits, uniforms and swimwear)” (the final example is reverse scored). For each question, participants respond on a Likert scale ranging between 1 (strongly disagree) and 5 (strongly agree). The PI20 has a possible range of 20–100, with lower scores reflecting better perceived face recognition ability. The PI20 has high reliability, Cronbach’s alpha = 0.92–0.96 (Matsuyoshi & Watanabe, 2021; Shah et al., 2015a, b) and shows moderate to high correlations with performance on face memory tests (Gray et al., 2017; Matsuyoshi & Watanabe, 2021; Shah et al. 2015a) and the Glasgow Face Matching Test (Shah et al. b), which draws on the same database of stimuli used in the current study. To ensure that the control sample did not include individuals with suspected face recognition problems, any participant who scored more than 2SDs above the control norms reported in Shah et al. (2015a, b) (a score of 66 or above) were excluded from the control sample.

The control sample for the DP group consisted of 25 participants (11 female, 14 male; *M*_age_ = 40.16*, *SD = 8.09). The control sample for the SR group consisted of 25 participants (8 female, 17 male; *M*_age_ = 38.80*, *SD = 7.54).

#### Materials and procedure

Individuals with DP and SRs completed the same matching tasks as used in Experiment 1. All data was collected online via the Testable.org website.

Prior to the experiment, all participants provided informed consent via an online consent form. This project was approved by the institutional Research Ethics Committee.

#### Statistical analyses

As in Experiment 1, SDT measures *A* and *b* were calculated for the matching task, and mean ratings in each condition were calculated for the trustworthiness, competence, and attractiveness judgements. Preliminary analyses once again revealed departures from normality in many variables (primarily due to skewed data). Levene’s test showed that the homogeneity of variance assumption was violated for a small number of variables in the analysis; where appropriate, follow up analyses on these variables were carried out using non-parametric tests.

We examined the potential difference between groups separately for the DPs and SRs, using ANCOVAs with age entered as a covariate, group (controls; DPs or SRs) as a between-subjects factor, and mask (mask; no mask) and sunglasses (sunglasses; no sunglasses) as within-subjects factors. As data from typical individuals has been reported in Experiment 1, we focussed specifically on the presence of main effects of group and interactions with group, which speak to the hypothesis that DPs and SRs show a different effect of masks or sunglasses than individuals with typical face processing skills. All the data from the study can be accessed at https://osf.io/m2ch8/?view_only=a5b8edc88bcc4d6ca3f7b9b56b57b0d6.

Finally, we used data collected in Experiment 1 to examine whether the effect of masks and sunglasses in the typical population correlated with self-reported face recognition ability (scores on the PI20).

### Results

#### Individuals with DP

**Fig. 5:**
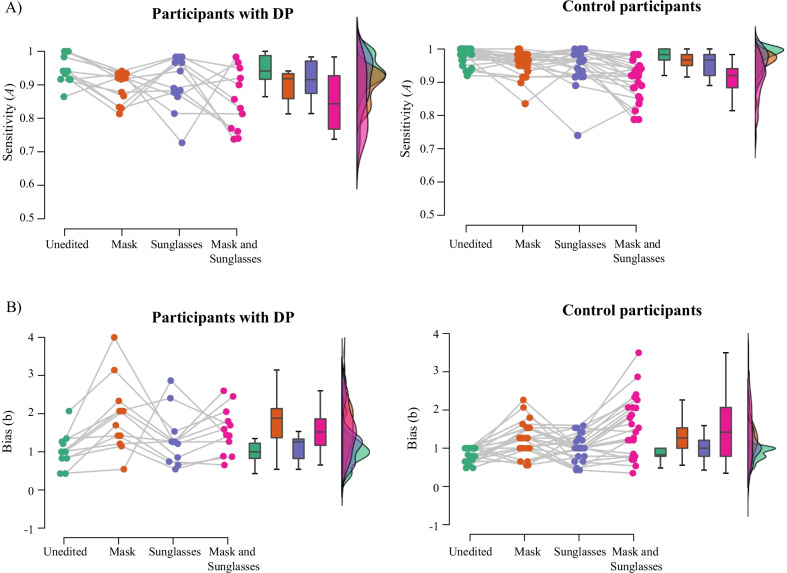
Raincloud plots of **A** discrimination (**A**) and **B**) bias (b) in the matching task for individuals with DP and the matched control group in each image condition. Coloured points show performance of each individual in each condition; box plots display median and upper/lower quartiles for each group in each condition, and histograms represent the distribution of data in each condition

Performance on the matching task for the DP group and the matched control group is shown in Figs. 5 (SDT measures) and 6 (Proportion correct across trial types). A 2 (group: controls, DPs) × 2 (mask: mask, no mask) × 2 (sunglasses: sunglasses, no sunglasses) ANCOVA on *A* values with age as a covariate revealed a main effect of group, *F*(1,34) = 13.47, *p* < .001, *ηρ*^2^ = .28. Averaged across conditions, controls (*M* = 0.95, SE = 0.01) outperformed DPs (*M* = 0.91, SE = 0.01).

**Fig. 6:**
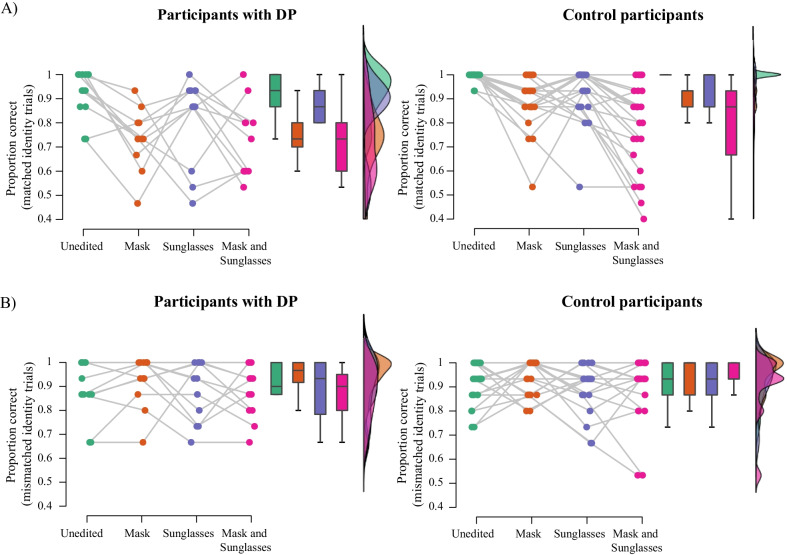
Raincloud plots of proportion correct responses in **A** matched identity and **B** mismatched identity trials in the matching task for individuals with DP and the matched control group in each image condition. Coloured points show performance of each individual in each condition; box plots display median and upper/lower quartiles for each group in each condition, and histograms represent the distribution of data in each condition

The sunglasses × group interaction was not significant, *F*(1,34) = 0.00, *p* = .986, *ηρ*^2^ = .00; nor was mask × group interaction, *F*(1,34) = 1.36, *p* = .252, *ηρ*^2^ = .04, or the mask × sunglasses × group interaction, *F*(1,34) = 0.32, *p* = .578, *ηρ*^2^ = .01.

An identical ANCOVA on *b* values revealed no significant main effect of group, *F*(1,34) = 3.94, *p* = .055, *ηρ*^2^ = .104, and no significant two-way interactions involving group, mask × group *F*(1,34) = 0.52, *p* = .477, *ηρ*^2^ = .02; sunglasses × group: *F*(1,34) = 1.46, *p* = .235, *ηρ*^2^ = 0.04. The mask × sunglasses × group interaction was significant, *F*(1,34) = 6.16, *p* = .018, *ηρ*^2^ = .153. Simple main effects analyses revealed that control participants showed a significant effect of masks on bias (specifically, an increase in conservative responding) regardless of whether faces were wearing sunglasses or not, *p*’s = 0.002. The DP group showed a significant effect of masks when faces were not wearing sunglasses, *p* = .032, but not when sunglasses were present, *p* = .546.

An ANCOVA exploring the effect of trial type on accuracy across groups revealed a main effect of group, *F*(1,34) = 12.45, *p* = .001, *ηρ*^2^ = .104. Similarly to the *A* analysis, controls (*M* = 0.91, SE = 0.02) outperformed DPs (*M* = 0.85, SE = 0.01). There were no significant interactions involving group, all *p*’s > 0.07.

Each analysis was repeated with the reduced (difficult) dataset; the pattern of results remained the same.

A 2 (group: controls; DPs) × 2 (mask: mask; no mask) × 2 (sunglasses: sunglasses; no sunglasses) × 2 (trial type: matched; mismatched) ANOVA on correct RT revealed that, on average, DPs were significantly slower than control participants to respond, *F*(1,34) = 5.18, *p* = .029, *ηρ*^2^ = .132; however, no other effects involving group were significant, all *p*’s > 0.09.

#### SRs

**Fig. 7:**
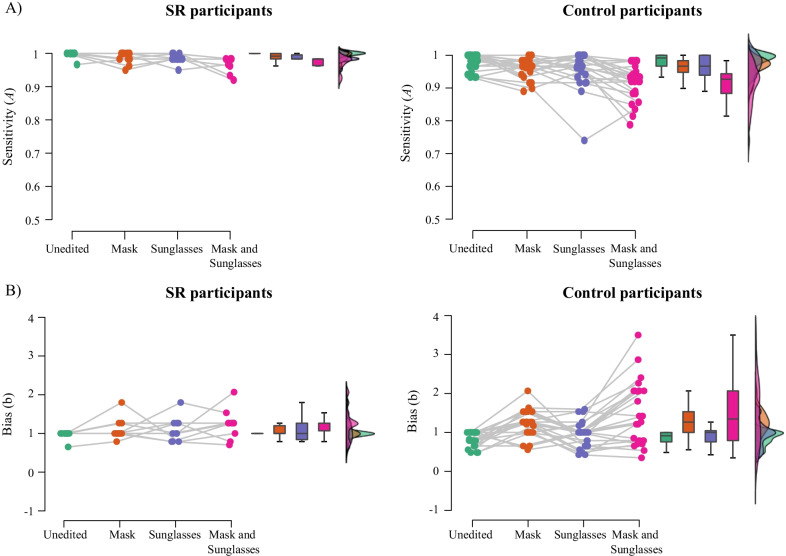
Raincloud plots of **A** discrimination (**A**) and **B** bias (b) in the matching task for SRs and the matched control group in each image condition. Coloured points show performance of each individual in each condition; box plots display median and upper/lower quartiles for each group in each condition, and histograms represent the distribution of data in each condition

Performance on the matching task for the SR group and the matched control group is shown in Figs. 7 (SDT measures) and 8 (Proportion correct across trial types). A 2 (group: controls, SRs) × 2 (mask: mask, no mask) × 2 (sunglasses: sunglasses, no sunglasses) ANCOVA on *A* values with age as a covariate revealed a main effect of group, *F*(1,32) = 6.30, *p* = .017, *ηρ*^2^ = .17. Averaged across conditions, SRs (*M* = 0.98, SE = 0.01) outperformed controls (*M* = 0.94, SE = 0.01).

**Fig. 8:**
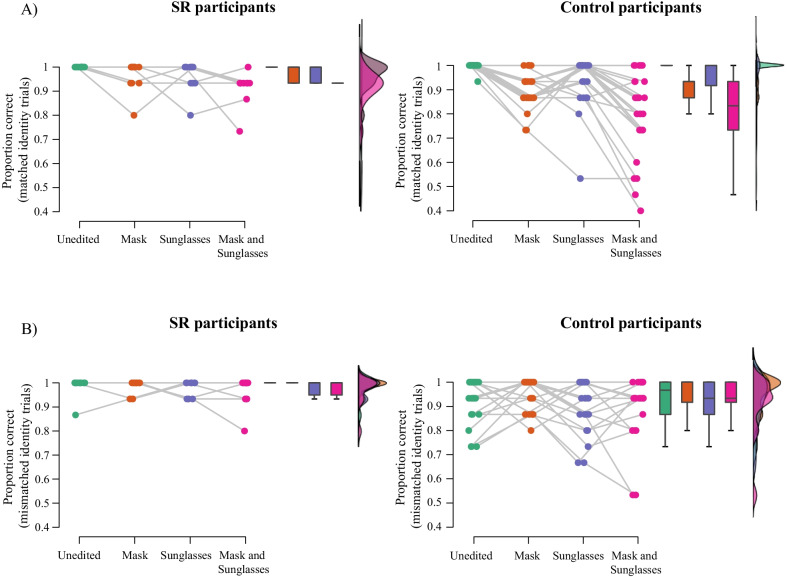
Raincloud plots of proportion correct responses in **A** matched identity and **B** mismatched identity trials in the matching task for SRs and the matched control group in each image condition. Coloured points show performance of each individual in each condition; box plots display median and upper/lower quartiles for each group in each condition, and histograms represent the distribution of data in each condition

The sunglasses × group interaction was not significant, *F*(1,32) = 2.06, *p* = .161, *ηρ*^2^ = .06; nor was mask × group interaction, *F*(1,32) = 1.48, *p* = .233, *ηρ*^2^ = .04, or the mask × sunglasses × group interaction, *F*(1.,32) = 0.56, *p* = .814, *ηρ*^2^ = .00.

An identical ANCOVA on *b* values revealed no significant main effect of group, *F*(1,32) = 0.00, *p* = .968, *ηρ*^2^ = .00. The sunglasses × group interaction was not significant, *F*(1,32) = 0405, *p* = .832, *ηρ*^2^ = .001, nor was the three way interaction between sunglasses, masks, and group, *F*(1,32) = 0.13, *p* = .719, *ηρ*^2^ = .00. The mask × group interaction was significant, *F*(1,32) = 5.59, *p* = .024, *ηρ*^2^ = .15. Simple main effects analyses revealed that control participants showed a significant effect of masks on bias (specifically, an increase in conservative responding), *p* < .001, whereas the SR group did not, *p* = .506.

An ANCOVA exploring the effect of trial type on accuracy across groups revealed a main effect of group, *F*(1,32) = 6.93, *p* = .013, *ηρ*^2^ = .18. Similarly to the *A* analysis, SRs (*M* = 0.96, SE = 0.02) outperformed controls (*M* = 0.86, SE = 0.01). There was also a significant interaction between trial type, mask, and group, *F*(1,32) = 6.80, *p* = .014, *ηρ*^2^ = .18. Simple main effects revealed that, as in Experiment 1, controls showed a significant effect of masks for matched identity trials, *p* < .001, but not mismatched identity trials, *p* = .292. By comparison, SRs did not show a significant effect of masks for either the matched or mismatched identity trials, *p*’s > 0.3.

Once again, each analysis was repeated with the reduced (difficult) dataset; the pattern of results remained the same.

A 2 (group: controls; SRs) × 2 (mask: mask; no mask) × 2 (sunglasses: sunglasses; no sunglasses) × 2 (trial type: matched; mismatched) ANOVA on correct RT did not show a significant main effect of group, *F*(1,32) = 3.84, *p* = .059, *ηρ*^2^ = .11. Both two-way interactions with group were significant: mask × group: *F*(1,32) = 17.77, *p* < .001, *ηρ*^2^ = .36; sunglasses × group: *F*(1,32) = 5.91, *p* = .021, *ηρ*^2^ = .16. The two-way interaction between mask and group was superseded by a significant three-way mask × group × trial type interaction, *F*(1,32) = 5.35, *p* = .027, *ηρ*^2^ = .14: simple main effects revealed that, while SRs were faster to respond to unmasked faces regardless of whether the faces were matched or mismatched, *p*’s < .005; control participants were faster for unmasked than masked faces in matched identity trials, *p* = .005, but not mismatched identity trials.

In sum, the effect of face coverings on face matching performance was relatively consistent across the three groups of participants. The key exception was matched identity trials, where SRs showed a reduced effect of masks compared to controls.

#### Self-reported face recognition ability and the effect of masks and sunglasses (control participants).

For each participant, we calculated the difference between performance with unedited faces and performance with masks, sunglasses, and both (mask effect, sunglasses effect, and combined effect, respectively) for each measure from the matching task (*A*, *b*, accuracy on matched and mismatched trials).

To examine the relationship between self-reported face recognition ability and face coverings, the mask, sunglasses, and combined effects for each measure were correlated with the PI20 scores for each participant. Correlations are displayed in Table 4. No significant correlations were found between the PI20 and any effect for *A*, *b*, or mismatched identity trials, *p*’s > 0.09. The mask effect for matched identity trials showed a small but significant correlation with the PI20, indicating that a larger mask effect (i.e., a bigger decline in accuracy between unedited images and images with masks) was associated with slightly poorer self-reported face recognition abilities.

**Table 4:** Correlations between self-reported face recognition ability (PI20) and the effects of masks, sunglasses, or both occlusions

	Mask effect (unedited—mask)	Sunglasses effect (unedited—sunglasses)	Combined effect (unedited—mask and sunglasses)
Sensitivity (*A*)			
Pearson’s *r*	.126	.142	− .045
*p*	*.148*	*.101*	*.605*
Bias (*b*)			
Pearson’s *r*	− .147	− .103	.115
*p*	*.090*	*.236*	*.185*
Accuracy (matched identity trials)			
Pearson’s *r*	**.179**	.147	− .126
*p*	*.038*	*.090*	*.146*
Accuracy (mismatched identity trials)			
Pearson’s *r*	− .042	.063	.023
*p*	*.629*	*.467*	*.796*

Bold cells indicate a significant correlation, *p* < .05. Italicised values represent the *p* values associated with the Pearson's *r*, regardless of significance

In sum, there was no relationship between the detrimental effect of face coverings and most aspects of self-reported face recognition ability. Once again, the only exception was the effect of masks on matched identity trials, where a greater effect of masks correlated with poorer self-reported face recognition abilities.

### Discussion

Experiment 2 examined whether the effects of masks differ based on individual differences in face recognition ability. Individuals at the extreme ends of the face recognition ability spectrum (those with DP and SRs) did not show a disproportionate effect of masks or sunglasses on face matching performance compared to control participants. However, there were some minor differences between groups which reflected different patterns of responding: control participants responded more conservatively to masked than unmasked faces, whereas this tendency was not present in SRs, and was only present for masked faces with sunglasses in DPs. SRs also showed a reduced effect of masks when matching two faces of the same person.

There was little evidence that individual differences in self-reported face recognition ability within the typical population correlated with the effects of masks or sunglasses on most measures. The exception was accuracy on matched identity trials—smaller mask effects were associated with better face recognition ability. This is in line with the results from the SRs. Thus, we find little evidence to suggest that individual differences in the effects of masks on identification are associated with individual differences in face recognition ability more generally.

## General discussion

Over the past 18 months, the use of face masks has increased dramatically in Western countries. Previous research has established that masks may influence social judgements (Biermann et al., 2021; Marini et al., 2021) and face identification (Carragher & Hancock, 2020; Freud et al., 2020; Marini et al., 2021; Noyes et al., 2021); this study sought to examine whether these effects are consistent across time, and whether they are associated with individual differences in face recognition ability.

### Masks and face processing

In line with previous research (Carragher & Hancock, 2020; Graham & Ritchie, 2019; Noyes et al., 2021), our results indicate that face masks and sunglasses have a significant negative effect on face matching performance, which appears to be driven by a decrease in accuracy in same identity (match) trials, rather than different identity (mismatch) trials. In short, occlusions such as masks and sunglasses affect individuals’ ability to correctly identify when faces show the same person (sometimes referred to as “telling faces together”; Andrews et al., 2015); but they have a minimal effect on individuals’ abilities to reject mismatched pairs (“telling people apart”). One potential explanation for this result is the asymmetry of perceptual information required for the two judgements: it may be possible to discriminate between faces based on a single distinguishing feature (e.g., a difference in the eyes, or the nose, or the mouth); however, telling faces together requires some level of confidence that the whole face is the same.

When presented in isolation (i.e., masks or sunglasses alone), the effects of different facial coverings were almost identical in degree: when averaged across the timepoints measured in Experiment 1, accuracy between the two conditions differed by less than 3% for same identity trials (masks: 87%; sunglasses: 89.3%) and less than 2% for different identity trials (masks: 91.7%; sunglasses: 90.4%). The current findings are slightly different to the results from Noyes et al. (2021), who found a larger effect of face coverings than sunglasses, but they are in accord with research suggesting that unfamiliar face recognition relies on cues from across the whole face (Abudarham & Yovel, 2016; Gosselin & Schyns, 2001). Further, they indicate that these cues can be used somewhat flexibly in matching tasks, at least when participants are given unlimited time to respond.

It is noteworthy that we did not match the size of the occluded area across conditions: masks generally occluded a larger proportion of the face than sunglasses did (although this varied slightly depending on the style of masks and sunglasses edited onto each image). This implies that the location/specific features being occluded, as opposed to the size of the occlusion, is the important factor for identification. However, as we did not systematically vary the size of the masks and sunglasses on each face, it is unclear whether varying the size of the occlusions (e.g., oversized sunglasses; masks covering smaller areas of the face) would lead to different levels of performance. Further, given that masks always occluded multiple features (mouth and nose), it is unclear whether the negative effects on identification were driven by the absence of one or both features. Previous work found that the use of transparent masks (which still occluded the nose, but showed the mouth region) still impaired identification performance (Marini et al., 2021); future work may systematically vary the availability of these features (e.g., depicting masks worn underneath the nose) to determine the perceptual cues which drive the effects of masks.

The size of the effect of masks in Noyes et al.’s (2021) study was higher than in the current work (7.5%). Given the lack of effects of timepoint, it is unlikely that this difference arose due to our participants adjusting to face coverings over time—instead, it may reflect the fact that our images were edited to include occlusions, whereas theirs were more naturalistic and could have included more variability between face images. Alternatively, it may reflect some degree of familiarity with the faces, since participants viewed the same individuals several times in the ratings tasks before completing the matching task. Indeed, our baseline (unedited) accuracy levels were higher than Noyes et al. (94% in the current study, compared to 81.5% in Noyes et al.). However, average matching performance in the unedited condition was only slightly higher than the extended version of the Glasgow Unfamiliar Face Test (89.9% in Burton et al., 2010), suggesting that participants’ performance was not out of the normal range for standard tests of face matching. Furthermore, additional analyses on a subset of more difficult trials found similar results: the difference in accuracy between unedited and masked faces was 5.5% in the complete dataset, and 4.3% in the difficult dataset. Unsurprisingly, including both masks and sunglasses on the same face further impaired identification. However, performance remained well above chance levels for all conditions, suggesting that, at least for the GUFD stimuli, some level of identification is still possible when the main features of the face are occluded (see also Duchaine & Weidenfeld, 2003). This could be due to the presence of informative non-facial cues in these images—although two different images of each individual were used in the current study, the images were captured around 15 min apart (Burton et al., 2010), so external cues such as hairstyle were largely similar across image pairs.

The effects of masks on trait judgements were somewhat variable, and depended on the judgement being made and the presence of other face coverings (i.e., sunglasses). When compared to uncovered faces, the addition of a face mask led to higher ratings of competence, but not trustworthiness (c.f. Biermann et al., 2021). When compared to faces wearing sunglasses (which, in general, received lower ratings on all traits), the addition of a face mask did not affect judgements of competence, but resulted in lower judgements of trustworthiness. For attractiveness, masks had slightly different effects depending on the presence or absence of sunglasses; however, simply comparing masked and unmasked faces did not result in a significant change in ratings. As such, there is limited evidence that masks influence ratings of attractiveness.

The limited effects of masks alone on trustworthiness were surprising, given the results of previous research in this area (Biermann et al., 2021; Marini et al., 2021). However, Marini et al. (2021) only reported effects of masks on trustworthiness for a subset of the images in their sample, and Biermann et al. (2021) found that the effects of masks varied based on attitudes towards masks in their sample. Similarly, decisions to co-operate with mask-wearing or non-mask-wearing individuals can vary depending on the context and mask-wearing behaviour of an individual (Powdthavee et al., 2021). Taken together with the present findings, these studies suggest that the effects of masks on trustworthiness judgements and behaviours are highly variable, and depend on the context (both visual and social), the face being judged, and the individual doing the judging.

To our knowledge, this is the first study that has systematically examined the effects of face masks on attractiveness and competence. Our results do not support anecdotal claims about the use of masks to increase attractiveness (“maskfishing”); however, given the variable effects of masks on other trait judgements such as trustworthiness (Biermann et al., 2021; Marini et al., 2021), it would be advisable for future research to investigate the stability of these effects in different populations and with different stimuli. Masks (when presented in isolation) also increased ratings of competence. It is unclear why this effect occurred. One possibility is that high levels of pro-mask social media coverage (Lang et al., 2021) may have led to people making more positive trait attributions to individuals wearing masks, although it is unclear why this effect did not generalise to trustworthiness judgements.

Although the main focus of this research was the effects of masks, it is notable that our results also diverge from previous research which has found limited effects of sunglasses on judgements of attractiveness and competence (Graham & Ritchie, 2019). Once again, the differences may be attributable to the nature of the stimuli (edited vs naturalistic). It is likely that individuals select sunglasses which suit their face shape and enhance their appearance, particularly in images uploaded to the internet. This may have ameliorated the negative effects of sunglasses on attractiveness in Graham and Ritchie’s work.

It is difficult to judge whether the effects of masks and sunglasses on social judgements arises from a change in the perceptual cues available, or if it is a reflection of how people evaluate mask-wearers more generally. For example, the decrease in attractiveness and trustworthiness ratings for faces wearing both masks and sunglasses could reflect the fact that both upper and lower facial cues and symmetry information are obscured; alternatively, people wearing both sunglasses and masks could be perceived as trying to obscure their face. Further research comparing the effects of different occlusions (e.g., masks vs scarves or artificial occlusions, as in Fischer et al., 2012; Kret & de Gelder, 2012) could help identify the locus of these effects.

### Consistency of mask effects over time

The magnitude of mask (and sunglasses) effects did not differ significantly across the three timepoints. This suggests that the effects of masks are relatively stable over time, despite increasing levels of exposure. These conclusions are somewhat tempered by the cross-sectional nature of our sample: we were not able to compare whether the effects of masks varied over time within participants. It is possible that some individuals who experienced very high exposure to masks throughout the pandemic (or before) may have developed compensatory strategies that counteract the negative effects of masks on face recognition. However, as all the participants in the current study were living within the UK at the time of testing (and therefore were subject to similar mask regulations at similar times), and the final sample had been interacting with individual in masks for up to a year, it is reasonable to conclude that increased exposure to masks over time has not dramatically decreased their impact on recognition. Consequently, in environments where face matching or identification is of high importance, it may be necessary to enact alternative COVID-19 protective measures (e.g., clear face shields or barriers) to enable the safe removal of masks when required.

In relation to social judgements, it may be that time or exposure to masks is less influential than attitudes that people carry about masks and mask-wearers (Biermann et al., 2021). Consequently, it is important that future research take these factors into account when considering how masks impact face perception more broadly.^3^

### Individual differences in face recognition

The effects of masks on identification did not vary systematically between typical perceivers and individuals with very poor face recognition abilities (DP). These results are somewhat unexpected, as some previous research has shown that some individuals with DP focus more on the mouth region (which is covered by face masks) when looking at faces (e.g., Bobak et al., 2017). However, individuals with DP also report using a variety of compensatory strategies when identifying faces (e.g., hairstyle), and eye-tracking studies have also shown a greater focus on external features of the face in DP compared to controls (Schmalzl et al., 2008; Schwarzer et al., 2007). Many of these cues remained available in the current stimuli, so it is possible that DPs were able to compensate for lack of information in the lower face by attending to external features (e.g., hairstyle).

Alternatively, it is possible that the DPs in this sample processed faces in a similar manner to controls. DP is a heterogeneous condition, and many individuals with DP do not show atypical face perception skills (e.g., Bate et al., 2019c; Chatterjee & Nakayama, 2012; Dalrymple et al., 2014). On a group level, our results indicated that the DP participants performed worse than controls and SRs overall in the matching task. However, the perceptual deficit displayed by the DPs in the control condition (equivalent to 0.52 SDs below the control mean) was relatively small compared to their level of impairment on face memory tasks (> 2 SDs below control performance). This could indicate relatively typical face perception skills in this sample—in that case, it is unsurprising that the effects of masks were in line with those displayed by control participants.

One way to discriminate between these explanations (compensatory strategies vs typical face perception) is to examine reaction times. Our analyses of RT indicated that DPs were significantly slower than controls when completing the matching task overall, suggesting that at least some participants with DP may have been employing slow compensatory strategies. However, it is important to note that we did not tell participants to focus on speed when responding. Further, the mode of response (using a mouse to select the correct option on screen) is not optimal for recording reaction times. As such, the RT data is primarily useful as an indication that control participants were not engaging in speed-accuracy trade-offs. Future research may wish to extend on the current work by using tasks more suited to measurement of reaction times, and recording eye movements while participants match partially occluded faces.

SRs showed a slightly reduced mask effect compared to typical perceivers when matching two images of the same face; however, we did not find a significant difference in the effects of masks for typical perceivers and SRs in the primary (*A*) analysis. The RT analysis also confirmed that SRs were slower to match masked than unmasked faces. These findings are in line with previous work which suggests that SRs are still susceptible to face processing biases and limitations that are present in typical perceivers (Bate et al., 2019a, 2020). However, our results diverge from those of Noyes et al. (2021), who found a different pattern of performance for SRs compared to typical perceivers. Noyes et al. also noted no differences in bias between their groups, whereas SRs in the current study showed a reduced effects of masks on bias compared to controls.

Given the similarities between the paradigms in the two studies, it is difficult to account for these differences. One possible explanation is that the SRs in the current study showed extremely high accuracy: it is possible that ceiling effects may have attenuated the differences between groups. However, the same patterns of difference in bias were also present for a reduced (more difficult) subset of trials. Another difference between the studies is that our sample was smaller than the sample employed by Noyes et al., which could have limited our power to identify small effects. Nonetheless, the differences between control participants and SRs in their study was relatively small, suggesting that the effects of masks for SRs are not dramatically different to those in the typical population. Consequently, organisations which employ SRs in face-matching tasks should be aware that even individuals who are extremely proficient at face processing may be negatively affected by face coverings. Once again, this suggests that alternative protective measures may be required in situations where identification is of high importance.

The absence of group-level differences in mask effects between DPs or SRs, and typical perceivers was supported by the lack of correlation between self-reported face recognition ability and mask (and sunglasses) effects for most measures in the matching task. Like the SRs, the exception was the mask effect for matching identity trials. Notably, this is the same measure that appears to underpin the mask effect in the main analysis—taken together, this indicates that masks make it more difficult to identify when two images show the same person, but these effects are reduced in those with better face recognition. This is somewhat unexpected, as previous research with SRs has not indicated that they excel specifically at certain trial types (Bate et al., 2018).

While the results from the matching identity trials offers some explanation for the variability in the mask effects, it is important to note that the effects are quite small. Further, the same correlations do not appear in other measures of performance, such as *A*. This is not simply due to a disconnect between self-report and objective measures of face recognition ability: there was a small but significant negative relationship between PI20 scores and both sensitivity and overall accuracy in the unedited faces condition of the matching task, in line with the findings of Shah et al. (2015a, b). Consequently, it is unclear why the effects of masks on face processing vary so substantially between individuals.

It may be that other individual differences in face processing can account for this variability: for example, some evidence suggests that there is inter-observer variability in preferred first fixation locations to faces. In other words, some individuals preferably look to the middle of the face (around the nose) first, while others look higher on the face (Peterson & Eckstein, 2013). While these differences themselves do not relate to face recognition, performance is better when participants can fixate in their “preferred” location (Peterson & Eckstein, 2013). Masks (or other occlusions such as sunglasses) may prevent observers from employing their preferred viewing strategy, thus affecting recognition performance.

Alternatively, variability in the effects of masks may also be attributed to other factors such as attitudes: for example, masks could create “in-groups” and “out-groups” (Powdthavee et al., 2021), which can affect face recognition (Bernstein et al., 2007). Mask effects could also vary depending on the availability and use of different compensatory information (e.g., hair, body, voice) by different individuals. Further work examining the effects of masks on person perception (as opposed to just face perception) and using more naturalistic stimuli (e.g., videos) could help to clarify when the effects are more apparent, and which individuals are most likely to be negatively affected.

### Conclusions

In conclusion, face coverings such as masks and sunglasses have a modest but significant impact on face processing, which is consistent over time. The effects of masks and sunglasses are relatively similar for face identification tasks (i.e., matching). However, the relationship between masks and sunglasses is more complex for other social judgements; consequently, research and policy decisions related to masks should take into account a variety of contextual factors, rather than simply trying to quantify the difference between masked and unmasked faces. Given the stability of effects over time, further effort (e.g., training) or the use of clear masks/visors may be required in situations where face identification is crucial. Based on our results, the overall effect of masks is driven by difficulty matching images that show the same person; this effect is ameliorated slightly in individuals with better face recognition ability. Consequently, training programmes seeking to minimise mask effects should consider focusing on strategies designed to help “tell people together”, as opposed to discriminating between different individuals.

## Data Availability

The datasets analysed in the current study is available in the OSF respository, https://osf.io/m2ch8/?view_only=a5b8edc88bcc4d6ca3f7b9b56b57b0d6. The stimuli used in this study cannot be made publicly available due to restrictions in publishing the images from the Glasgow Unfamiliar Face Database (Burton et al., 2010). A copy of the stimuli or a fully programmed version of task (programmed in Testable.org format) is available from the corresponding author on reasonable request.

## Footnotes

1 In this paper, we use the term “masks” to include face coverings which cover the nose and mouth region, encompassing surgical-style masks as well as non-medical cloth face coverings.

2 Initial analyses compared the DP and SR groups to the entire sample of individuals who completed Experiment 1; however, the differences in age distributions and extreme violations of the homogeneity of variance assumption mean the conclusions from these analyses may be unreliable. Nonetheless, it is noteworthy that the pattern of results using different control groups is almost identical.

3 Data on mask-wearing and participants’ attitudes towards people who do/do not wear masks in different environments was collected for timepoints 2 and 3 and is available in the dataset associated with this paper. However, as data was not available for all timepoints, it was not analysed in the current study.
